# Postpartum depression in maternal thyroidal changes

**DOI:** 10.1186/s13044-022-00124-6

**Published:** 2022-03-29

**Authors:** Paula Michele da Silva Schmidt, Aline Longoni, Ricardo Tavares Pinheiro, Adriano Martimbianco de Assis

**Affiliations:** Center of Health Science, Postgraduate Program in Health and Behavior, Universidade Católica de Pelotas - UCPel, Pelotas, RS 96015-560 Brazil

**Keywords:** Thyroid, Thyroid peroxidase antibodies, Pregnancy, Postpartum depression, Perinatal depression

## Abstract

Evidence in the literature has suggested that there may be an association between thyroid antibodies and depression during pregnancy and in the postpartum period. Thus, this study aims to conduct a systematic review on the prevalence of postpartum depression (PPD) in women with thyroid abnormalities during pregnancy or in the postpartum period. For this review, we used four databases (PubMed, Lilacs, Scielo, and Scopus). Fifteen studies were selected; one study used a case–control design, four used a cross-sectional design and ten utilized prospective cohort designs. All studies were restricted to up to 1 year postpartum, and 46.7% focused on a period between immediate postpartum and 6 months postpartum. Estimates of the prevalence of PPD in pregnant women with thyroid disorders ranged between 8.3% and 36.0%. For follow-up studies, the cumulative incidence of self-reported depression from the primary episode in the first postpartum year was 6.3% in a high-city survey. Although some authors consider the status of positive anti-TPO antibodies to be a possible marker of vulnerability to depression , it is not yet possible to conclude whether thyroid function in the pregnancy-puerperal cycle is involved with the development of PPD.

## Introduction

Depression, one of the most frequent mental disorders after childbirth, is considered a severe and current health problem due to its high prevalence and the negative impact that it has on both the mother's health and the development of her child [1]. The postpartum period, considered one of the most complex experiences of human life [2], is characterized by an emotional vulnerability that, associated with physiological, psychological, social, and cultural changes, promotes the appearance of maternal mental disorders. PPD is defined as a mood disorder that usually manifests itself in the first four weeks after delivery and usually reaches its maximum intensity in the first six months after delivery.

Epidemiological studies on PPD suggest a multiplicity of risk factors involved in its genesis, among which are endocrinopathies [3, 4]. Thyroid disorders are the endocrine diseases that have been most researched to identify an association with postpartum depression. During pregnancy, the maternal immune system undergoes many changes to accommodate the development of the fetus [5] and tries to return to its prepregnancy state in the postpartum period. These modifications include changes in the production of autoantibodies that target thyroid antigens, such as thyroid peroxidase (anti-TPO) [6]. Anti-TPO is the most common type of thyroid autoantibody found in euthyroid individuals and is associated with various forms of thyroid dysfunction [7].

Several studies have shown a positive association between the thyroid peroxidase antibody and the development of mood disorders, with a high prevalence of positive anti-TPO antibodies among patients with bipolar and unipolar disorders. However, this association between positive thyroid antibody and postpartum depression has not been replicated in other studies. Menna et al. [8] and Le Donne et al. [9] concluded that there is insufficient evidence to confirm an association between PPD and postpartum thyroiditis or positive anti-TPO antibodies (in euthyroid women who did not develop postpartum thyroiditis).

The relationship between thyroid dysfunction and PPD cannot yet be considered consistent [10, 11] and requires further study. Therefore, the present study aims to assess the potential of thyroid biomarkers as predictors for the development of PPD through a systematic review. Our working hypothesis is that one or more thyroid markers can predict the risk of developing PPD in pregnant/puerperal women, and these markers may be related to worse disease.

## Methods

### Review question

Can thyroid markers predict the risk of developing PPD in pregnant/puerperal women?

### Inclusion and exclusion criteria

As an inclusion criterion, the publication should contain original data, and the research must be carried out with humans, not including literature reviews, editorials, perspectives, letters, commentaries, and abstracts from meetings. No other exclusion criteria, such as language, year limit, sample sizes, or diagnostic tools for PPD, were used.

### Search strategy

This study was based on a systematic review of scientific articles published in indexed journals until the date of January 24, 2022. The PubMed, Lilacs (Latin American and Caribbean Literature in Health Sciences) SciELO (Scientific Electronic Library Online), and Scopus databases were searched, according to Table 1. For the outcome (thyroid changes), the terms “thyroid dysfunction”, “thyroid hormones”, “TPO protein, human”, “thyroid peroxidase antibody”, “hypothyroidism” and “hyperthyroidism” were used. As terms of exposure (PPD), the terms “depression, postpartum” and “perinatal depression” were used. In the Lilacs and SciELO databases, the terms “postpartum depression” and “postnatal depression” were used to determine if they were present in the articles, according to Health Sciences Descriptors (DeCS).

**Table 1:** Structured search strategy carried out in databases. The search followed the structure of each database

Databases	MeSH term and entry terms
PubMed	(depression, postpartum[MeSH Terms] OR Perinatal Depression[MeSH Terms] AND Thyroid Hormones[MeSH Terms] OR TPO protein, human[MeSH Terms] OR Hypothyroidism[MeSH Terms] OR Thyroid Peroxidase Antibody[MeSH Terms] OR Thyroid Dysfunction[MeSH Terms] OR Hyperthyroidism[MeSH Terms]) Filters: Clinical Trial, Randomized Controlled Trial, Humans
Lilacs	("Postpartum Depression" [Palavras] or "Pregnancy" [Palavras] and "Thyroid" [Palavras])
Scielo	("Postpartum Depression") OR ("Perinatal Depression") AND ("Thyroid Peroxidase Antibody") OR ("Thyroid Dysfunction")
Scopus	“thyroid dysfunction” OR “thyroid hormones” OR “TPO protein, human” OR “thyroidperoxidase antibody”, “hypothyroidism” AND “hyperthyroidism” “depression, postpartum” AND “perinatal depression”

All references were managed in EndNote X7 software (Thomson Reuters, New York, NY, US). Initially, duplicate references were excluded. Titles and abstracts were independently screened based on the aforementioned inclusion and exclusion criteria by two reviewers (PMSS and AL). The screened lists were compared, and in case of disagreement, a consensus was reached by discussion. When a consensus was not achieved, a third reviewer decided if the article should be included (AMA). After the initial screening of titles and abstracts, full articles were evaluated by the same two reviewers. In addition to an electronic search, the reviewers also performed a hand search in the reference lists of all included studies. Predefined data collection worksheets were used for the data extraction of each selected publication. This systematic review followed the PRISMA statements, with some adjustments [12].

### Data extraction

**Fig. 1:**
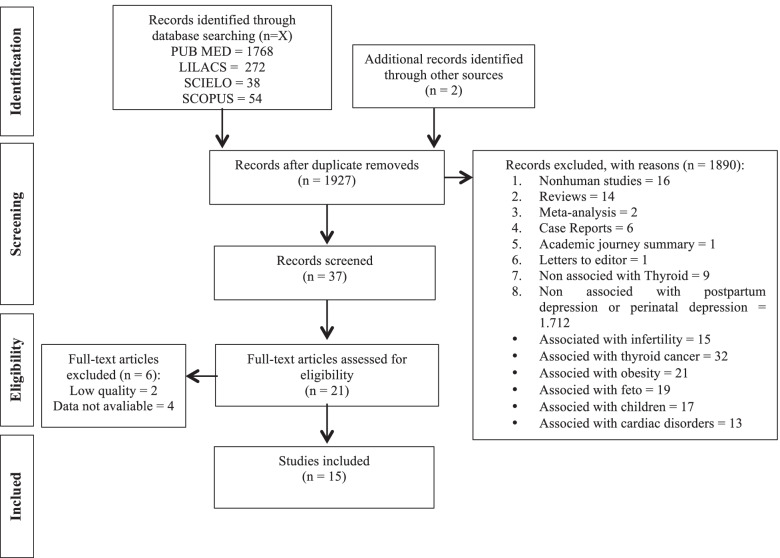
Flow chart analysis of the articles selected in the PubMed, LILACS, Scielo and Scopus databases (January 24, 2022)

The titles and abstracts of the studies were initially analyzed. For the full evaluation, publications with original results on the prevalence or incidence of PPD in women/pregnant women with previous thyroid evaluation were selected (Fig. 1). A secondary search was also carried out in the bibliographic list of the articles initially evaluated to identify other important references not captured by the initial search.

In addition to data on thyroid assessment and PPD, information was collected on methodological aspects that could have some influence on the results of suspected or diagnosed cases of PPD, such as the study design, sociodemographic profile of the population evaluated, gestational and postpartum period used and blood analysis method and criteria.

The process of searching, extracting, and tabulating the selected articles was performed independently by two reviewers (PMSS and AL) to be submitted to descriptive analysis. Cases of disagreement were handled as described above. Both reviewers also manually searched the reference section of each relevant article and independently assessed and collected all other articles identified as eligible. A meta-analysis of the data was not feasible, given the absence of agreement in the literature to date.

### Statistical analysis

We chose not to meta-analyze data from eligible studies because significant heterogeneity exists across studies in terms of the assays used to measure anti-TPO and the cutoffs used to determine anti-TPO (i.e., normal or abnormal levels), the methods used to define depression, and the timing of assessment for both TPO-AB status and depression. Consistent timing in the measurement of TPO-AB and thyroid hormones during pregnancy is required for valid meta-analysis given the changing immune milieu of this period of life [13, 14].

## Results

Our initial searches yielded 1768, 272, 38, and 54 studies in the PubMed, Lilacs, SciELO, and Scopus databases, respectively (Fig. 1). Twenty-one full text articles were examined as they were thought to contain data that could address our objective (associations between anti-TPO during pregnancy or the puerperium or postnatal depression). However, only 15 original articles suggested the presence, in the full text, of data on the frequency of PPD and thyroid assessment [9, 15–28]. The selected articles were published between 1992 and 2019.

The characteristics and results of the studies included in this review can be found in Table 2. The sample sizes in these studies ranged from 31 [15] to 1075 [16]. One study used a case–control design [17], four used a cross-sectional design [9, 18–20], and ten utilized prospective cohort designs [15, 16, 21–28].

**Table 2:** Summary of selected studies on maternal thyroid disorder and postpartum depression

Author(s)/ Year/ Study design	Thyroid evaluation	Postpartum period studied	Instrument	Research Population	% PPD	Results	Conclusion
Harris et al. [21] 1992 Prospective Cohort Study	TPO-AB was assessed as a dichotom ous variable The study used a MS-AB assay to assess for TPO-AB status TPO-AB + (≥ 525 U/ml) at 16 weeks gestation	8,12,20, and 28 weeks postpartum	EPDS (≥ 13), HADS (≥ 11), and HAM-D (≥ 15)	145 antibody-positive women and 229 antibody-negative women delivering between August 1987 and December 1989	Not applicable The estimated antibody prevalence in depressed women was significantly higher (16 1%, 95% confidence interval 12 1 to 19–8) than that in women without depression (9 3%)	Follow up of 110 antibody-positive and 132 antibody-negative women showed significantly greater depression by research diagnostic criteria in antibody-positive women (47%) than antibody-negative women (32%) regardless of thyroid dysfunction. Antibody-positive women showed higher mean scores for depression on the Hamilton (6.01 v 3.89, *p* = 0.0002), Edinburgh (7.45 v 5.92, *p* = 0.031), and hospital depression scales (4.95 v 3.79, *p* = 0.003)	Depressive symptoms are associated with positive thyroid antibody status in the postpartum period
Pop et al. [22] 1993 Prospecti ve cohort study	To investigate the association s between Anti-TPO-AB and the occurrence of postpartum depression. TPO-AB was assessed as a dichotomous variable The study used a MS-AB assay to assess for TPO-AB status TPO-AB + (any detectable titers of MS-AB at 32 weeks gestation)	Starting at 4 weeks postpartum, and at 6 week intervals until 34 weeks postpartum	.Depression was assessed using the Research Diagnostic Criteria developed by Spitzer et al.,without knowing the results of biochemical thyroid function tests	293 women at 32 weeks' gestation	Tthe incidence of depression developing during the postpartum period of assessment was 20.8% (61 women). Twenty women (6.8%) met the criteria for major depression, and 41 women (13.9%) for minor depression. Of the 27 MsAb positive women at 32 weeks' gestation, 9 subsequently developed depression during the postpartum period, compared to 52 of the 266 MsAb negative women at 32 weeks' gestation	At 32 weeks' gestation there were 27 (9.2%) women with elevated microsomal antibody titres. Compared with microsomal antibody-negative women at 32 weeks' gestation, these women had an RR of 20 for developing postpartum thyroid dysfunction and an RR of 1.7 for developing postpartum depression	Women with elevated microsomal antibody titres during gestation are particularly at risk for postpartum thyroid dysfunction, but only have a slightly increased risk for postpartum depression
Lazarus et al. [23] 1996 Prospective Cohort Study	Anti-TPO was assessed as dichotom ous variable anti-TPO + (≥ 19.6 kIU/l) measured at 16 weeks of gestation	Starting at the first month of postpartum and at monthly intervals for the first year of postpartum	Symptoms of depression were measured by a symptom questionnaire. Starting at the first month of postpartum and at monthly intervals for the first year of postpartum	474 women were recruited from a district hospital in South Wales over a two year period	Not applicable	Significantl y more TPO-AB + women reported depressive symptoms vs. TPOAB(-) women at the first month of postpartum (no data reported; *p* < 0.007)	Although postpartum thyroiditis (PPT) is usually transient, this condition, and the euthyroid antibody-positive state, may be associated with significant symptomatology, including an increased incidence of minor to moderate depression
Kent et al. [18] 1999 Cross-sectional Study	Thyroid dysfunction was defined as TSH or fT4 outside the adult reference range (TSH 0,34–4,8 mU/l and fT4 10–19 pmol/l); Microsomal antibody (MsAb + > 1: 400; TPOAb + > 49 kIU/l; Thyroid receptor antibodies (TRAb) with TSH < 0,34 mU/l using a reference range < 10 units/l; Thyroid stimulating antibodies (TSI) < 1,3 units/l)	25 weeks postpartum (Range: 20–41 weeks)	General Health Questionnaire (GHQ28) (Goldberg & Hillier, 1979); Composite International Diagnostic Interview (CIDI-A) padronizado e computadorizado (Janca et al., 1994); DSM-III-R criteria (The American Psychiatric Association, 1987.)	748 Women who were Caucasian, aged 20– 45 years and 4·5–5·5 months postpartum	The prevalence of PPTD in the participants was 11·5% (95% CI 9·2–13·8%). The percentage of depression for anti-type positive women was not evaluated	The 6 month point prevalence rates of depression, generalized anxiety disorder and panic disorder and/or agoraphobia were 9·4%, 1·4% and 3·1%, respectively No relationship was found between PPTD status and the diagnosis of current depression or between thyroid antibody status and current depression. In women who were diagnosed as anxious at the time of assessment, the number of anxiety symptoms was higher in the PPTD group (*P* < 0·05)	This study has shown a high prevalence of postpartum thyroid dysfunction but there was no difference in the clinical and psychiatric signs and symptoms between cases and controls
Kuijpens et al. [24] 2001 Prospective Cohort Study	TPO-AB + (> 50 U/ml)	4,12,20,28, 36 weeks postpartum	Syndromal diagnosis for depression (major or minor) using the RDC diagnostic criteria	310 Women were recruited from local midwifery practices or at the Obstetrics Department of St. Joseph Hospital in the Netherlands	59% TPOAB + and 38% TPO-AB(-)	TPOAB + group had significantl y more women develop postpartum depression than the TPO-AB(-) group (59% vs. 38%; *p* = 0.03)	The presence of TPOAbs during gestation is associated with the occurrence of subsequent depression during the postpartum period and as such can be regarded as a marker for depression
Ruschi et al. [19] 2009 Cross-sectional Study	TSH, free T4, anti-TPO	Between 31^st^ and 180^th^ postnatal days	EPDS, 11/12 cutoff point	292 women from Public Health Units in the city of Vitória/ES in Brazil	115 (39.4%) women with PPD Group with changes thyroid: 36%	There was no statistically significant difference in the PPD frequency between patients with and without thyroid disorder (x^2^ = 0.131; *p* = 0.717)	The frequency of PPD was high, with no association between PPD and thyroid changes
Lambrinoudaki et al. [20] 2010 Cross-sectional Study	Free T4, free T3, TSH, anti-TG, and anti-TPO measured at admission until the 4th postpartum day	Admission up to 6 weeks postpartum	PQB, on admission and on Days 1 to 4 postpartum EPDS, on Day 4 and at 6 weeks postpartum	57 native Greek women from a Hospital at Aretaieion University with gestational ages of 35 to 38 weeks	Not applicable	Free T3 and free T4 in the prepartum period were negatively correlated with PBQ scores in the first postpartum week. As for thyroid antibodies, no association was found with mood scores	The findings indicate an association between the occurrence of postpartum mood disorders and prenatal thyroid function. Lower levels of free T3 and free T4 are associated with an increased incidence of mood disorders in the first postpartum week
Albacar, et al. [17] 2010 Cross-sectional Study	Free T4, TSH, anti-TPO, PCR analyzed 48 h postpartum	Between 24–48 h postpartum, 8 weeks and 32 weeks postpartum	EPDS, cutoff point 9/10	1053 women of Spanish origin in the postpartum period and with no previous history of depression	Among the 1053 women, 87 (8.3%) were depressed. Although 152 women (14.4%) had high levels of anti-TPO and slightly elevated TSH concentrations with normal free T4	No association was found between thyroid function and PPD. Thyroid dysfunction was not associated with CRP concentrations that were outside normal levels. Although thyroid function was not associated with PPD, when the entire study population was considered, it was observed that women with anti-TPO + had an increased risk of hypothyroidism (OR 5.54)	It was concluded that thyroid function at 48 h postpartum does not predict the risk of PPD. However, it may be that the anti-TPO positivity observed, together with other hormonal and molecular factors, worsens thyroid function and that subsequently anti-TPO positivity may be associated with PPD
Bergink et al. [15] 2011 Longitudinal Study	Anti-TPO antibodies were quantified as immunological measures of AITD TSH and free T4 levels were also measured to assess clinical thyroid dysfunction	4 weeks and 9 months postpartum	Structural Clinical Interview for DSM-IV (SCID)	31 primiparous women from the community of the provinces of South Holland, Zealand and North Brabant. Without a psychiatric history diagnosed with postpartum psychosis	In the 4 weeks postpartum 19% of women with postpartum psychosis had AITD compared to controls (13%; OR = 2.78, 95% CI 1.08–7.17), and in 9-month postpartum 29% of women with postpartum psychosis had AITD compared to controls (13%; OR = 2.78, 95% CI 1.08–7.17), respectively	Patients with postpartum psychosis had a significantly higher rate of progression from subclinical AITD to clinical thyroid dysfunction. Specifically, of the patients with AITD at the 9-month follow-up, 67% had thyroid dysfunction compared to only 20% of the control group (OR = 8.00, 95% CI 1.23–52.25)	Women with postpartum psychosis are at higher risk not only for AITD but also for clinical thyroid failure. In addition, AITD represents a potentially strong etiological factor for the development of postpartum psychosis
Le Donne, Settineri and Benvenga [9] 2012 Cross-sectional Study	TSH, free T3, free T4, anti-thyroperoxidase (anti-TPO) and anti-thyroglobulin (anti-TG) antibodies	3 days postpartum	EPDS, MADRS, and TAS. Cutoff scores of 12 (EPDS), 15 (MADRS) and 61 (TAS) were used	74 Caucasian Italian women from a university hospital. The average age of women was 31.8 (range 20–44, median 31.5)	There is no % of PPD in women with thyroid dysfunction. The rates of women with abnormal EPDS, MADRS and TAS scores were similar (31%, 30% and 28.4%, respectively)	The alexithymitic individuals had lower T4, higher T3, lower T4free/T3free ratio and higher levels of anti-TPO or anti-TGT. Only anti-TPO and anti-TG were significantly higher in women at risk for PPD, but only at the EPDS cutoff values of 13 or 14. TAS was shown to be directly correlated with anti-TPO and FT3, and inversely with the T4free: T3free relationship, while EPDS correlated only with anti-TPO	It was concluded that the risk of PPD and alexithymia is directly associated with thyroid autoimmunity, and its association with serum thyroid hormones and the free T4/free T3 ratio follows opposite directions
Groer e Vaughan [25] 2013 Prospective Cohort Study	Anti-TPO and TSH dosages were performed, in addition to a specific physical examination and a checklist of thyroid symptoms developed by the authors was applied. Anti-TPO + > 20 IU/ml	During pregnancy and in the postpartum period. Follow-up during the 6 months postpartum	Perceived Stress Scale (PSS) and the Mood States Profile Questionnaire (POMS)	47 anti-TPO positive and euthyroid women agreed to continue the follow-up. A control group of anti-TPO negative women (*n* = 72) was randomly selected for follow-up	Of the total sample of pregnant women, 48 were diagnosed with PPD (6.8%). During the postpartum period, 13 women in the total sample had scores indicating for clinical PPD. These women were all referred Eight (61.5%) of the 13 were TPO positive	Pregnant women with positive anti-TPO had significantly more depressive symptoms and were more likely to score higher than 20 on the POMS scale than women with negative anti-TPO (*p* = 0.028). Anti-TPO positive women had significantly higher scores for depression, anger and total postpartum mood disturbance scores than anti-TPO negative women, regardless of the development of postpartum thyroiditis (*n* = 25)	The results suggest that the presence of positive anti-TPO antibodies in pregnant women and euthyroid mothers increases the possibility of negative dysphoric mood, and especially of depressive symptoms that cannot be explained by stress or demographic factors
Sylvén et al. [26] 2013 Population-based Cohort Study	TSH, free T4 and anti-thyroid peroxidase antibodies (anti-TPO)	Blood samples during EPDS delivery at 5 days, 6 weeks and 6 months after delivery	EPDS-Edinburgh Postnatal Depression Scale), cutoff point of 12 or more	347 Swedish women at Uppsala University Hospital, Sweden	After adjustment for previous psychiatric contact, smoking during pregnancy, prepregnancy BMI and sleep, TSH levels above 4.0 mU/L were associated with an increased risk of depressive symptoms at six months postpartum (OR 11.30 95% CI 1.93–66.11)	Among the 329 samples successfully analyzed for TSH, 21 women (6.4%) had levels above 4.0 mU/L. Anti-TPO was analyzed in 248 samples and nine of the women tested had high levels of anti-TPO (3.6%). There was no significant association between PPD and TSH levels at five days or six weeks postpartum	The study suggests a screening tool to identify individuals at risk of developing PPD. However, other works must test this concept in a prospective scenario
Pedersen et al. [29] 2016 Prospective Cohort Study	17-estradiol, progesterone and thyroxine binding globulin (TBG). Variables of primary interest: total T4, free T4 and TBG, associated with thyroid variables of secondary interest: TSH, total T3, free T3 and uptake of T3 resin (T3U)	4 home visits, 2 during pregnancy (31–33 weeks and 35–36 weeks) and two postpartum (6 and 12 weeks)	EPDS; IDATE; MADRS; Hamilton Anxiety Scale (HRDS); and the modified MINI Plus 5.0.0	A cohort of 199 euthyroid women recruited from a public health antenatal clinic located in North Carolina	There is no % of PPD in women with thyroid dysfunction. Based on MINI-Plus interviews, 22 (11.1%) of subjects met DSM-IV criteria for major depression or RDC minor depression during late pregnancy (35–36 weeks), and 24 (12.1%) met criteria postpartum (week 12)	When analyzed in isolation, the level of free T4 was a less strong but still significant predictor of depression and anxiety (*p* < 0.05), while TBG levels were a significant or almost significant predictor of most classifications. Total T4, TBG and trauma history were significant individual predictors of syndromic depression during the study period (*p* < 0.05) in models with a single predictor. In models that combine each with a history of trauma and major depression, free T4 and TBG were not significantly predictive of depression or anxiety, and free T4 was also not a significant predictor of syndromic depression	It was shown that lower concentrations at the end of pregnancy of an endocrine variable sensitive to estrogen, TBG, predict perinatal syndromic depression. This result is particularly significant when there is a history of trauma and major depression
Wesseloo et al. [16] 2018 Prospective Cohort Study	anti-TPO, TSH and free T4. A level > 20 IU/ml was defined as positive anti-TPO	10^th^-12^nd^ weeks of gestation	Self-reported PPD was defined using the following validated cutoff scores in the EPDS: 1^st^ trimester ≥ 11; 2^nd^ and 3^rd^ trimester ≥ 10; postpartum ≥ 13	1075 pregnant women with follow-up during pregnancy until one year postpartum	The cumulative incidence of self-reported depression in the first episode in the first postpartum year was 6.3%	A positive anti-TPO state was associated with an increased risk of self-reported depression of single onset at four months postpartum (adjusted OR 3.8; 95% CI 1.3–11.6), but not in other postpartum periods studied. The prevalence of PPD decreased after four months postpartum in the positive anti-TPO group, but remained constant in the negative group. The association between anti-TPO status and self-reported single-onset. depression at 6 weeks, 8 months and 12 months after delivery was not significant	Women with increased anti-TPO during pregnancy have a higher risk of self-reported depression from the first episode. The longitudinal pattern of self-reported depression in the postpartum group in the positive anti-TPO group was similar to the typical course of anti-TPO in the postpartum. This suggests overlap in the etiology of PPD and autoimmune thyroid dysfunction. It was concluded that thyroid function should be assessed in women with PPD
Zhang et al. [28] 2019 Prospective Cohort Study	Serum measurement of TSH and free T4 at three days (3d) and four weeks (4 s) postpartum	3 days and 4 weeks postpartum	Chinese version of the EPDS to assess postpartum depression at three days (3d) and four weeks (4w) postpartum	96 pregnant women recruited from a single hospital in China	The incidence of depression at 3d and 4w was 14.58% and 7.29%, respectively	There was no significant difference in the occurrence of PPD between TSH groups > 2.5 mUI/L and TSH ≤ 2.5 mUI/L. TSH and free T4 levels did not correlate significantly with EPDS at 3 days or with EPDS at 4 weeks. The incidence of PPD decreased by 4 weeks. In the EPDS follow-up assessment, we found that 10 of the 14 individuals who were initially diagnosed with PPD recovered spontaneously. This shows that TSH > 2.5 with high EPDS scores in 3 days after delivery should not be an indication for prophylactic use of thyroid hormone medication	The prenatal TSH level cannot predict the occurrence of PPD. Just as depression and TSH > 2.5 in three days postpartum should not be an indication for prophylactic use of thyroid hormone medication

Of these studies, seven reported on the relationships between anti-TPO during pregnancy and postnatal depression [16, 20–25], and five investigated the links between anti-TPO during the postpartum period and postnatal depression [9, 17–19, 26].

Of these, one study used a cutoff value of 19.6 kIU/l [23], two studies each utilized cutoffs of 20 IU/ml [16, 25], and one utilized 27 IU/ml [17], 34 IU/ml [9], 49 kIU/l [18], and 50 U/ml [24]. One study used a cutoff value of 525 U/ml of microsomal antibodies [21], and one study identified women as anti-TPO (TPO-AB +) if they detected any level of microsomal antibodies at 32 weeks of gestation [22]. Two studies also examined TPO-AB as a continuous measure [9, 17].

Estimates of the prevalence of PPD in pregnant women with thyroid disorders ranged from 8.3% [17] to 59.0% [24]. For follow-up studies, the cumulative incidence of self-reported depression from the first episode in the first postpartum year was 6.3% in a high-city survey (*n* = 1075) [16]. The incidence of depression at 3 days postpartum and 4 weeks postpartum was 14.58% and 7.29%, respectively [28]. Among other findings, women in the postpartum period (24–48 h) with positive anti-TPO had a five times greater risk of hypothyroidism [17]. Clinical thyroid dysfunction occurred in 19% of patients with postpartum psychosis compared to only 3% of the control group [15].

Anti-TPO and anti-TG levels were significantly higher in women at risk for PPD [9]. In a study by Groer and Vaughan [25], anti-TPO positive women had significantly higher scores for depression, anger, and total scores of mood disturbance postpartum than anti-TPO negative women, regardless of the development of postpartum thyroiditis (*n* = 25).

In another study, after adjustment for previous psychiatric contact, smoking during pregnancy, prepregnancy BMI and sleep, TSH levels above 4.0 mU/L were associated with an increased risk of depressive symptoms at six months postpartum [26]. Lower concentrations of TGB at the end of pregnancy also proved to be a strong predictor for perinatal syndromic depression, as well as a history of trauma [27].

The postnatal period in which maternal depressive symptoms were assessed varied between the selected studies (Table 3). Although all studies were restricted to up to 1 year postpartum, eight studies (53,3%) covered some period between the immediate postpartum period and 6 months postpartum [9, 18–20, 25–28], and seven publications (46,7%) carried out a maternal mental health assessment after 6 months [15–17, 21–24]. A follow-up after 1 year of the postpartum period was not performed by any of the studies.

**Table 3:** Instruments for diagnosis/screening of postpartum depression

Study	PPD diagnostic tool	Cutoff values	Evaluated period
Harris et al. 1992 [21]	EPDS HADS HAM-D	EPDS ≥ 13 HADS ≥ 11 HAM-D ≥ 15	8,12,20, and 28 weeks postpartum
Pop et al. 1993 [22]	RDC	Not applicable	Starting at 4 weeks postpartum, and at 6 week intervals until 34 weeks postpartum
Lazarus et al. 1996 [23]	Symptom questionnaire	Not Applicable	Starting at the first month of postpartum and at monthly intervals for the first year of postpartum
Kent et al. 1999 [18]	GHQ28 CIDI-A HAM-A HAM-D	GHQ28: caseness cutoff of 4 CIDI-A ≥ 5 HAM-A > 14 HAM-D > 17	25 weeks postpartum (Range: 20–41 weeks)
Kuijpens et al. 2001 [24]	RDC	Not applicable	4,12,20,28, 36 weeks postpartum
Ruschi et al. 2009 [19]	EPDS	EPDS > 11/12	Between 31 and 180 days postnatal
Lambrinoudaki et al. 2010 [20]	PQB EPDS	PQB 8,2 EPDS 11	PQB: on admission and on Days 1 to 4 postpartum EPDS: on Day 4 and 6 weeks postpartum
Albacar, et al. 2010 [17]	EPDS	EPDS > 9/10	Between 24–48 h postpartum, 8 weeks and 32 weeks postpartum
Bergink et al. 2011 [15]	SCID	Not included	4 weeks and at 9 months after delivery
Le Donne, Settineri and Benvenga 2012 [9]	EPDS MADRS TAS	EPDS > 12 MADRS > 15 TAS > 61	3 days postpartum
Groer and Vaughan 2013 [25]	PSS POMS	PSS not included POMS not included	During pregnancy and in the postpartum period during the 6 months postpartum
Sylvén et al. 2013 [26]	EPDS	EPDS ≥ 12	5 days, 6 weeks and 6 months after delivery
Pedersen et al. 2016 [29]	EPDS STAI MADRS HAM-A	Not included. Searched average and maximum and minimum values	4 home visits, 2 during pregnancy (31–33 wk and 35–36 wk) and 2 postpartum (6 and 12 wk)
Wesseloo et al. 2018 [16]	EPDS	1° trimester ≥ 11 2° and 3° trimester ≥ 10 Postpartum ≥ 13	First postpartum year: 6 weeks, 8 months and 12 months postpartum
Zhang et al. 2019 [28]	EPDS	Not applicable .Applied a linear relationship in the time	3 days and 4 weeks postpartum

*EPDS* Edinburgh Postnatal Depression Scale, *HADS* Hospital and Anxiety Depression Scale, *HAM-D* Hamilton Rating Scale for Depression, *RDC* Research Diagnostic Criteria [30], *GHQ28* General Health Questionnaire [31], *CIDI-A* Composite International Diagnostic Interview, *HAM-A* Hamilton Anxiety Rating Scale, *PQB* Podromal Questionnaire Brief version, *SCID* Structured Clinical Interview for DSM-IV, *MADRS* Montgomery-Asberg Depression Rating Scale, *TAS* Toronto Alexithymia Scale, *PSS* Perceived Stress Scale, *POMS* Profile of Mood States, *STAI* State portion of Speilberger State-Trait Anxiety Inventory

Concerning the detection instruments used for PPD screening or diagnosis (Table 3), nine studies (60,0%) used the Edinburgh Postnatal Depression Scale (EPDS), of which they made exclusive use of the EPDS 5 surveys (33,3%) [16, 17, 19, 26, 28]. The Research Diagnostic Criteria, RDC, was exclusively used in two publications (13,3%) [22, 24], and in only one publication (6,7%), the clinical interview (SCI) was used to characterize PPD [15].

The cutoff values for the EPDS also varied between studies from 9 to more than 12 points (Table 3). Only one publication used different cutoff points for the gestational trimesters and the postpartum period: 1^st^ trimester ≥ 11, 2^nd^ and 3^rd^ trimester ≥ 10 and postpartum ≥ 13 [16].

## Discussion

From this systematic review of the literature to clarify the relationship between maternal thyroid changes and postpartum depression, based on our search criteria, it was observed that the studies on the subject are heterogeneous in terms of study size, population studied, design (prospective, case–control, transversal), psychometric scale, and the evaluation of thyroid hormones (different analysis methods and different cutoff points). However, for some authors, the status of thyroid peroxidase antibodies has become considered a marker of vulnerability to depression. It is observed that studies have been concerned with assessing PPD and thyroid changes throughout the gestational period and in the postpartum period through longitudinal studies. Most studies followed the participants over a certain period, with periodic measurements of postnatal depressive symptoms, thus obtaining an estimate of the incidence of the condition. The studies that showed a relationship between PPD and thyroid function suggested that thyroperoxidase antibodies (anti-TPO) may be a possible target in the search for a biomarker to predict the development of emotional disorders, including PPD [16, 17, 21, 23–25].

Ruschi et al. [19] and Kuijpens et al. [24] showed that the frequency of PPD was high, without an association between PPD and thyroid alterations. Multiple studies examining associations between thyroid hormones and depression during the perinatal period have suggested a link [20, 26, 27, 29, 32–34]. However, a consensus does not exist as to whether clinical syndromes of thyroid dysfunction (e.g., hyper- and/or hypothyroidism) are linked to depression in the perinatal period [23, 35–38].

Regarding TSH, our research shows few studies directly correlating TSH levels and PPD [26, 28]. Zhang et al. [28] found no significant difference in the occurrence of PPD between the TSH groups > 2.5 mUI/L and TSH ≤ 2.5 mUI/L. However, for serum T4, a meta-analysis article with low heterogeneity conducted with population-based studies showed that serum T4 was positively correlated with depressed mood, while TSH was negatively associated with depressed mood [39]. A study by Sylvén et al. [26] suggested that there was no significant association between PPD and TSH levels at five days or six weeks after delivery. However, after adjustment for previous psychiatric contact, smoking during pregnancy, prepregnancy BMI and sleep, TSH levels above 4.0 mU/L were associated with an increased risk of depressive symptoms at six months postpartum.

The findings of our study showed heterogeneity in the methods used to investigate both thyroid alterations and PPD (Tables 2 and 3). According to Lewandowski et al. [33], when they evaluated baseline concentrations of free T4, free T3, and TSH at 30-min intervals in 110 healthy pregnant women, in a significant number of patients, the diagnosis of subclinical thyroid dysfunction could be misdiagnosed, not as a result of "disease", but as a result of physiological variation in TSH concentrations. Additionally, in 2021, Xing et al. [29] found that the TSH reference range was significantly influenced by sex, age, iodine intake, sample size, region and test methods and manufacturers. Therefore, for the reliability of the thyroid alteration in a sample, each laboratory must validate an appropriate TSH reference interval based on local conditions and based on the physiological variations of pregnant women, postpartum women and the postpregnancy period.

Lambrinoudaki et al. [22] investigated whether thyroid function within the normal range affects the incidence of postpartum mood disorders. The findings indicated an association between the occurrence of postpartum mood disorders and prenatal thyroid function. Within normal limits, lower levels of free T3 and free T4 were associated with an increased incidence of mood disorders in the first postpartum week.

However, Albacar et al. [27] did not observe any association between thyroid function and PPD. All women who scored 9/10 on the EPDS at 8 weeks and 32 weeks postpartum were defined as likely cases of major depression. Among the 1053 women evaluated in the study, 8.3% were depressed. Although 14.4% had high levels of anti-TPO and slightly elevated TSH concentrations with normal free T4, no association was found between thyroid function and PPD. It was concluded that thyroid function at 48 h after delivery does not predict the risk of PPD. However, it may be that the observed anti-TPO positivity worsens thyroid function and that subsequent anti-TPO positivity may be associated with PPD, requiring additional investigations at multiple postpartum intervals. For Le Donne et al. [18], the risk of PPD and alexithymia is directly associated with thyroid autoimmunity. The risk of postpartum depression and alexithymia had a significant correlation with positive anti-TPO, suggesting that these mood disorders may have neurobehavioral consequences of an autoimmune attack (because of the anti-TPO circulation in the CSF and its possible cross-reactivity with brain autoantigens) [18, 32].

After analysis, we found that higher prevalence and incidence rates of depression and/or more severe complaints of depression have been reported in antithyroperoxidase antibody (TPOAb)-positive women by some authors [16, 21, 23–26], while others could not demonstrate such an association [17–20, 26]. Sylven et al. [26] evaluated depressive symptoms during each trimester and at four postpartum moments (6 weeks, 4 months, 8 months, and 12 months). A positive anti-TPO state was associated with an increased risk of self-reported depression of a single onset at four months postpartum but not at other postpartum time points. The longitudinal pattern of self-reported postpartum depression in the positive anti-TPO group was similar to the typical course of anti-TPO in the postpartum period. This suggests an overlap in the etiology of PPD and autoimmune thyroid dysfunction. By analyzing these results, we can assess thyroid function in women with PPD. Similarly, the results of Groer and Vaughan [25] suggest that the presence of positive anti-TPO in pregnant women and euthyroid mothers increases the possibility of a negative dysphoric mood, especially of depressive symptoms that cannot be explained by stress or demographic factors.

It is noteworthy that among the studies included in this work, the reference value for anti-TPO positivity varied; for example, levels above 19.6 KIU/L [23] and serum levels above 50 IU/mL [24]. For the reference values ​​for TSH, the most indicated for the reliability of anti-TPO positivity is to compare the patient’s anti-TPO value with that of the local laboratory reference. However, the studies found do not refer to adjustment for this possible confounding factor.

For the PPD detection or screening instruments, we found that the most used scale was the Edinburgh Postnatal Depression Scale (EPDS), with cutoff values between 9 and more than 12 points (Table 3); nevertheless, in some studies, there was the use of other associated scales, such as the Montgomery-Asberg Depression Rating Scale, the Toronto Alexithymia Scale, the Hamilton Anxiety Scale, the Modified MINI Plus 5.0.0., the Perceived Stress Scale and the Mood States Profile Questionnaire [17–19, 21, 22], and even when used alone, the cutoff point differed between studies. The standardization of a gold diagnostic method is essential to guarantee the validity of the diagnosis of PPD in a population sample. The absence of this standardization in the studies found should be considered a limitation for the correct diagnosis of PPD.

Another limitation that should be considered in the studies addressed by this systematic review is the influence of the social conditions of pregnant women with PPD, since the development of PPD is also influenced by social factors, and these factors can have a more significant impact than a slight increase in anti-TPO. According to Zhang et al. [28], the most cited risk factors for PPD in the literature over the last 5 (five) years were lack of family or partner support, unplanned pregnancy, family or personal history of psychiatric illness, low education and being a minor. The research also concludes that social and emotional factors have more influence on the prevalence of PPD than economic factors.

Indeed, dysregulation of various endocrine systems has been implicated in the pathophysiology of both antenatal and postnatal depression [40, 41]. Research suggests that the etiology of perinatal depression involves a combination of social [42, 43], psychological [44, 45], and biological factors [40, 41, 46].

In summary, the studies comprising this systematic review suggest that associations may exist between anti-TPO-positive status during gestation and postpartum depression. However, further studies are needed that consider the aforementioned limitations and analyze different stages of the period because Anti-TPO fluctuates throughout gestation and the postpartum period [13, 14].

## Conclusion

**Fig. 2:**
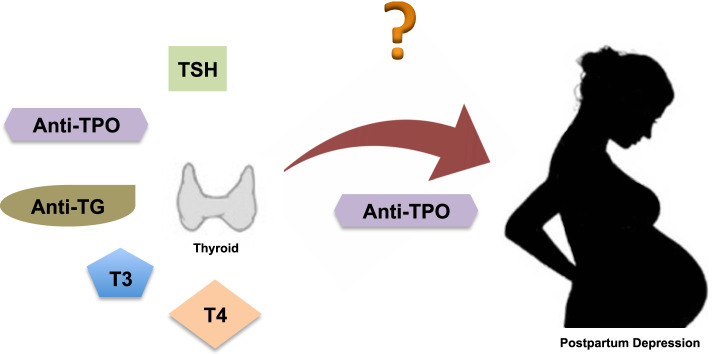
Our main finding is that there is no definition in the literature about the function of the thyroid gland and the development of PPD in pregnant/puerperal women. However, most studies that showed a relationship between PPD and thyroid function suggest that thyroperoxidase antibodies (anti-TPO) may be a possible target in the search for a biomarker to predict the development of PPD

After analyzing these results, it is clear that the association between anti-TPO antibodies and PPD was previously examined with contradictory results. The studies carried out are heterogeneous in terms of study size, population studied, design (prospective, case–control, transversal), psychometric scale, and anti-TPO measures (different analysis methods and different cutoff points). Although some authors consider the status of positive anti-TPO antibodies to be a possible marker of vulnerability to depression (Fig. 2), it is not yet possible to conclude which are the mechanisms of thyroid function involved in the pregnancy-puerperal cycle and PPD.

## Data Availability

None.

## References

[1] Gelaye B, Rondon MB, Araya R, Williams MA (2016). Epidemiology of maternal depression, risk factors, and child outcomes in low-income and middle-income countries. Lancet Psychiatry.

[2] Yim IS, Tanner Stapleton LR, Guardino CM, Hahn-Holbrook J, Dunkel SC (2015). Biological and psychosocial predictors of postpartum depression: systematic review and call for integration. Annu Rev Clin Psychol.

[3] Ghaedrahmati M, Kazemi A, Kheirabadi G, Ebrahimi A, Bahrami M (2017). Postpartum depression risk factors: A narrative review. J Educ Health Promot.

[4] Theme Filha MM, Ayers S, da Gama SG, Leal MoC (2016). Factors associated with postpartum depressive symptomatology in Brazil: The Birth in Brazil National Research Study, 2011/2012. J Affect Disord.

[5] Zenclussen AC (2013). Adaptive immune responses during pregnancy. Am J Reprod Immunol.

[6] Balucan FS, Morshed SA, Davies TF (2013). Thyroid autoantibodies in pregnancy: their role, regulation and clinical relevance. J Thyroid Res..

[7] Galofre JC, Haber RS, Mitchell AA, Pessah R, Davies TF (2010). Increased postpartum thyroxine replacement in Hashimoto's thyroiditis. Thyroid.

[8] Meena M, Chopra S, Jain V, Aggarwal N (2016). The Effect of Anti-Thyroid Peroxidase Antibodies on Pregnancy Outcomes in Euthyroid Women. Journal of clinical and diagnostic research. JCDR.

[9] Le Donne M, Mento C, Settineri S, Antonelli A, Benvenga S (2017). Postpartum Mood Disorders and Thyroid Autoimmunity. Front Endocrinol.

[10] Abalovich M, Amino N, Barbour LA, Cobin RH, De Groot LJ, Glinoer D (2007). Management of thyroid dysfunction during pregnancy and postpartum: an Endocrine Society Clinical Practice Guideline. J Clin Endocrinol Metab.

[11] Keshavarzi F, Yazdchi K, Rahimi M, Rezaei M, Farnia V, Davarinejad O (2011). Post partum depression and thyroid function. Iran J Psychiatry.

[12] Moher D, Liberati A, Tetzlaff J, Altman DG, Group P (2009). Preferred reporting items for systematic reviews and meta-analyses: the PRISMA statement. J Clin Epidemiol.

[13] Glinoer D, Riahi M, Grün JP, Kinthaert J (1994). Risk of subclinical hypothyroidism in pregnant women with asymptomatic autoimmune thyroid disorders. J Clin Endocrinol Metab.

[14] Stagnaro-Green A, Roman SH, Cobin RH, El-Harazy E, Wallenstein S, Davies TF (1992). A prospective study of lymphocyte-initiated immunosuppression in normal pregnancy: evidence of a T-cell etiology for postpartum thyroid dysfunction. J Clin Endocrinol Metab.

[15] Bergink V, Kushner SA, Pop V, Kuijpens H, Lambregtse-van den Berg MP (2011). Prevalence of autoimmune thyroid dysfunction in postpartum psychosis. Br J Psychiatry.

[16] Wesseloo R, Kamperman AM, Bergink V, Pop VJM (2018). Thyroid peroxidase antibodies during early gestation and the subsequent risk of first-onset postpartum depression: A prospective cohort study. J Affect Disord.

[17] Albacar G, Sans T, Martin-Santos R, Garcia-Esteve L, Guillamat R, Sanjuan J (2010). Thyroid function 48h after delivery as a marker for subsequent postpartum depression. Psychoneuroendocrinology.

[18] Kent GN, Stuckey BGA, Allen JR, Lambert T, Gee V (1999). Postpartum thyroid dysfunction: Clinical assessment and relationship to psychiatric affective morbidity. Clin. Endocrinol. (Oxf).

[19] Ruschi GEC, Chambô-filho A, A. SJVL, Zandonade E, Mattar R (2009). Alteração tireoidiana: um fator de risco associado à depressão pó s-parto?. Rev Bras Saude Mater Infant.

[20] Lambrinoudaki I, Rizos D, Armeni E, Pliatsika P, Leonardou A, Sygelou A (2010). Thyroid function and postpartum mood disturbances in Greek women. J Affect Disord.

[21] Harris B, Othman S, Davies JA, Weppner GJ, Richards CJ, Newcombe RG (1992). Association between postpartum thyroid dysfunction and thyroid antibodies and depression. BMJ.

[22] Pop VJ, de Rooy HA, Vader HL, van der Heide D, van Son MM, Komproe IH (1993). Microsomal antibodies during gestation in relation to postpartum thyroid dysfunction and depression. Acta Endocrinol. (Copenh).

[23] Lazarus JH, Hall R, Othman S, Parkes AB, Richards CJ, McCulloch B (1996). The clinical spectrum of postpartum thyroid disease. QJM.

[24] Kuijpens JL, Vader HL, Drexhage HA, Wiersinga WM, van Son MJ, Pop VJ (2001). Thyroid peroxidase antibodies during gestation are a marker for subsequent depression postpartum. Eur J Endocrinol.

[25] Groer MW, Vaughan JH (2013). Positive thyroid peroxidase antibody titer is associated with dysphoric moods during pregnancy and postpartum. J Obstet Gynecol Neonatal Nurs.

[26] Sylvén SM, Elenis E, Michelakos T, Larsson A, Olovsson M, Poromaa IS (2013). Thyroid function tests at delivery and risk for postpartum depressive symptoms. Psychoneuroendocrinology.

[27] Pedersen C, Leserman J, Garcia N, Stansbury M, Meltzer-Brody S, Johnson J (2016). Late pregnancy thyroid-binding globulin predicts perinatal depression. Psychoneuroendocrinology.

[28] Zhang L, Li C, Wu S, Wang L, Qiao C (2019). Maternal thyroid function during late pregnancy is not a risk factor for postpartum depression. Psychiatry Res.

[29] Pedersen CA, Johnson JL, Silva S, Bunevicius R, Meltzer-Brody S, Hamer RM (2007). Antenatal thyroid correlates of postpartum depression. Psychoneuroendocrinology.

[30] Spitzer RL, Endicott J, Robins E (1978). Research diagnostic criteria: rationale and reliability. Arch Gen Psychiatry.

[31] Goldberg DP, Hillier VF (1979). A scaled version of the General Health Questionnaire. Psychol Med.

[32] Abou-Saleh MT, Ghubash R, Karim L, Krymski M, Bhai I (1998). Hormonal aspects of postpartum depression. Psychoneuroendocrinology.

[33] Ijuin T, Douchi T, Yamamoto S, Ijuin Y, Nagata Y (1998). The relationship between maternity blues and thyroid dysfunction. J Obstet Gynaecol Res.

[34] Saleh ES, El-Bahei W, El-Hadidy MA, Zayed A (2012). Predictors of postpartum depression in a sample of Egyptian women. Neuropsychiatr Dis Treat.

[35] Basraon S, Costantine MM (2011). Mood disorders in pregnant women with thyroid dysfunction. Clin Obstet Gynecol.

[36] Lucas A, Pizarro E, Granada ML, Salinas I, Sanmarti A (2001). Postpartum thyroid dysfunction and postpartum depression: are they two linked disorders?. Clin. Endocrinol. (Oxf).

[37] Pop VJ, de Rooy HA, Vader HL, van der Heide D, van Son M, Komproe IH, Essed GG (1991). Postpartum thyroid dysfunction and depression in an unselected population. N Engl J Med.

[38] Walfish PG, Meyerson J, Provias JP, Vargas MT, Papsin FR (1992). Prevalence and characteristics of post-partum thyroid dysfunction: results of a survey from Toronto. Canada J Endocrinol Invest.

[39] Williams MD, Harris R, Dayan CM, Evans J, Gallacher J, Ben-Shlomo Y (2009). Thyroid function and the natural history of depression: findings from the Caerphilly Prospective Study (CaPS) and a meta-analysis. Clin Endocrinol (Oxf).

[40] Meltzer-Brody S (2011). New insights into perinatal depression: pathogenesis and treatment during pregnancy and postpartum. Dialogues Clin Neurosci.

[41] Serati M, Redaelli M, Buoli M, Altamura AC (2016). Perinatal major depression biomarkers: A systematic review. J Affect Disord.

[42] Beck CT (2001). Predictors of postpartum depression: an update. Nurs Res.

[43] Lancaster CA, Gold KJ, Flynn HA, Yoo H, Marcus SM, Davis MM (2010). Risk factors for depressive symptoms during pregnancy: a systematic review. Am J Obstet Gynecol.

[44] Bunevicius R, Kusminskas L, Mickuviene N, Bunevicius A, Pedersen CA, Pop VJM (2009). Depressive disorder and thyroid axis functioning during pregnancy. World J Biol Psychiatry.

[45] Zeng Y, Cui Y, Li J (2015). Prevalence and predictors of antenatal depressive symptoms among Chinese women in their third trimester: a cross-sectional survey. BMC Psychiatry..

[46] Skalkidou A, Hellgren C, Comasco E, Sylvén S, Poromaa IS (2012). Biological aspects of postpartum depression. Women’s Heal.

